# Connexin43 in Post-Surgical Peritoneal Adhesion Formation

**DOI:** 10.3390/life12111734

**Published:** 2022-10-28

**Authors:** Jia Wang Chua, Moogaambikai Thangaveloo, Debbie Xiu En Lim, Leigh E. Madden, Anthony R. J. Phillips, David L. Becker

**Affiliations:** 1 Lee Kong Chian School of Medicine, Nanyang Technological University Singapore, Singapore 308232, Singapore; 2 Skin Research Institute Singapore, Singapore 308232, Singapore; 3 School of Biological Sciences, Auckland University, Auckland 1142, New Zealand

**Keywords:** gap junctions, connexins, inflammation, fibrosis, peritoneal adhesions

## Abstract

Objective: Post-surgical peritoneal adhesions are a serious problem for the quality of life and fertility. Yet there are no effective ways of preventing their occurrence. The gap junction protein Cx43 is known to be involved in fibrosis in several different organs and disease conditions often associated with inflammation. Here we examined the Cx43 dynamic expression in an ischemic button model of surgical adhesions. Methods: Using the mouse ischemic button model, Cx43 antisense was delivered in Pluronic gel to attenuate Cx43 expression. The severity of button formation and immunofluorescence analysis of Cx43 and TGF-β1 were performed. The concentration of tissue plasminogen activator via ELISA was also performed. Results: As early as 6 h after button formation, the Cx43 levels were elevated in and around the button and some weak adhesions were formed. By 24 h Cx43 levels had increased further and adhesions were more defined. At 7 days the adhesions were much more robust, opaque, and vascularized, requiring blunt or sharp dissection to break them. Cx43 antisense attenuated its upregulation and, reduced the number and severity of adhesions that formed. Conclusion: Targeting Cx43 after surgical procedures may be a potential therapeutic strategy for preventing adhesion formation or at least reducing their severity.

## 1. Introduction

In response to injury, a tissue needs to repair itself. This process involves four overlapping phases: haemostasias, inflammation, proliferation, and tissue remodelling [1]. Perturbation of these finely-tuned processes can lead to fibrosis and excessive extracellular matrix (ECM) deposition [2,3,4]. These are the processes also observed in pathological adhesion formation [2].

Gap junction dynamics play a pivotal role in the wound-healing process [2,5,6,7]. Moreover, hemichannels (HC) provide a pathway for the release of paracrine messengers promoting inflammation [8]. Since little is known about the role of connexins in peritoneal adhesion formation, understanding their role in fibrosis-related diseases may shed some light on their contribution to adhesion formation as the processes are similar between both aetiologies.

An elevation of Cx43 protein is observed in fibroblasts in the wound edges of human diabetic foot ulcers and streptozotocin-induced diabetic rats [9]. Conversely, fibroblasts harvested from hypertrophic scars or keloids have reduced Cx43 protein levels compared to normal skin [10]. In a recent study of gingival wounds, reduced Cx43 HC was shown to promote rapid and scarless healing [11]. Collectively, this suggests that Cx43 is essential for fibroblast homeostasis, maintaining a balance between proliferation, migration, and the production of ECM.

Treatment of skin wounds in mice with Cx43 antisense oligodeoxynucleotides (Cx43asODN) improves several aspects of wound healing [12]. These include reduced inflammation and an increase in transforming growth factor-β1 (TGF-β1), promoting myofibroblast differentiation, and increased hydroxyproline content [13]. Enhanced migration of fibroblasts was also observed in vitro when hemichannels and gap junctions were inhibited with connexin mimetic peptides [14,15].

In the heart, specialized fibroblasts can derive from differentiated fibroblasts stimulated by the release of TGF-β, cytokines, ECM, and growth factors [16,17,18,19,20]. TGF-β1-induced cardiac fibroblast differentiation into myofibroblasts can be driven by overexpression of Cx43 or inhibited by its reduction [21]. This was suggested to be due to the competitive binding between Smad2/3 and Cx43 to microtubules [22]. These findings indicate that Cx43 is involved in cardiac fibrosis.

Tubulointerstitial fibrosis is one of the hallmarks of chronic kidney disease (CKD) found in diabetic patients [2]. It is characterised by the mesenchymal transition of epithelial cells into myofibroblasts, which progressively leads to increased ECM deposition, fibrotic scar formation and deterioration of renal function [23]. High glucose-mediated TGF-β1 release from human proximal tubule cells induced epithelial-mesenchymal transition, resulting in fibrosis [24,25]. Cx43 expression was upregulated in the kidney of CKD in both humans and rodents [2,26]. Inhibition of Cx43 expression in Cx43^+/−^ transgenic mice or through Cx43asODN treatment in CKD models significantly reduced cell adhesion markers, monocyte infiltration and interstitial renal fibrosis [26].

These findings of disease-related fibrogenesis collectively suggest that high levels of Cx43 are associated with inflammation and fibrosis [9,12,13,21,27]. During adhesion formation, fibrin deposits facilitate the invasion and proliferation of fibroblasts [28] which then differentiate into myofibroblasts, increase ECM deposition and contribute to the development of adhesions [29].

Here the role of Cx43 in post-surgical peritoneal adhesion formation was explored. Our results demonstrate that an elevation of Cx43 protein occurred during the early phase of adhesion formation (6 and 24 h post-surgery). Attenuating the Cx43 elevation by applying Cx43asODNs reduced the extent of adhesion formation and fibrosis, suggesting a new therapeutic target against post-surgical peritoneal adhesion formation.

## 2. Materials and Methods

### 2.1. Animals and Ethics Approval

All animal procedures were performed with the approval of the Institutional Animal Care and Use Committee of the Animal Research Facility of Nanyang Technological University, (A0372). For each experiment, four to ten C57BL/6 male mice, approximately 7–12 weeks old were bred under pathogen-free conditions and maintained on a 12-h light-dark cycle with a standard rodent diet and water-supplied ad libitum. A detailed number of animals used for each experiment is described in figure legends.

### 2.2. Ischaemic Button Model of Post-Surgical Peritoneal Adhesions

Ischaemic buttons (IB) were created to induce peritoneal adhesion formation as previously described by Sandoval et al. (2016) [30] with slight modifications (Figure S1). Mouse fur around the incision site was removed before returning them to their cages for at least 24 h. Mice were anaesthetized with isoflurane (1–3% *v*/*v*) and placed on a heating mat. A midline incision site was sterilised with 7.5% providone-iodine solution and a midline laparotomy of 4 cm was performed to facilitate exposure of the parietal peritoneum. Each IB was created by placing a 4/0 non-absorbable silk suture at the base of ~3 mm of the sidewall parietal peritoneum grasped with a needle holder. Two IBs, spaced at least 1 cm apart, were created on the peritoneum wall of each side of the midline. Vehicle or treatment was added, and the laparotomy site was closed in two layers, the inner layer with simple interrupted polyglycolic acid absorbable 6.0 sutures and the outer layer using clips (Figure S1) Mice were euthanatized with CO_2_ at 6 and 24 h post-surgery for early phase adhesion formation and at 7 days post-surgery for late phase formation. The mice received a subcutaneous injection of buprenorphine for postoperative analgesia (0.1 mL per 10g of animal weight).

### 2.3. Studying the Role of Cx43 during Peritoneal Adhesion Formation

Mouse Cx43 antisense oligodeoxynucleotide, Cx43asODN (5′-GTAATTGCGGCAGGAGGAATTGTTTCTGTC-3′) was administered to the IB peritoneal adhesion to investigate the role of Cx43 in adhesion formation [12]. Two treatment formulations were applied to each button. First, 25 μL of 300 μM of Cx43asODN in 30% Pluronic^®^ F127 (PG) was topically delivered to each of the 4 buttons. Secondly, 400 μL of 300 μM of Cx43asODN in 20% PG was applied to the left and right flank of the peritoneal cavity. These formulations were delivered as a single application of treatment. A vehicle-only group (20% PG) acted as a control.

### 2.4. Peritoneal Lavage

3 mL of cold phosphate-buffered saline (PBS) was injected into the peritoneal cavity and gently agitated for 1 min. Approximately 2 mL of the fluid was recovered. Samples with blood contamination were omitted. Cellular debris was removed by microcentrifugation at 9000× *g* for 15 min at 4 °C and samples were analysed to determine the concentration of tissue plasminogen activator (tPA) and plasminogen activator inhibitor (PAI-1) present. An enzyme-linked immunosorbent assay (ELISA) was performed according to the manufacturer’s instructions (Molecular Innovations, Novi, MI, USA). Samples that had a coefficient of variation values of more than 15% were excluded. Detected concentrations were normalised using total protein content determined from a bicinchoninic acid assay performed on the same sample (ThermoFisher Scientific, Waltham, MA, USA).

### 2.5. Macroscopic Assessment of Peritoneal Adhesions

An inverted U-shaped incision was made across the peritoneal cavity for necropsy and adhesions in each animal were evaluated according to established scoring matrices. These matrices assessed peritoneal adhesions based on the extent, involvement, severity, and strength (Table S1) [31,32,33,34]. Three blinded observers scored each mouse, and their scores were averaged. Scores were represented as a percentage of the maximum score for each parameter.

### 2.6. Histology and Immunostaining

Tissue samples were processed into paraffin sections, hematoxylin and eosin (H&E) and immunofluorescence staining were performed as described in our previous work [35]. Briefly, paraffin tissue sections were rehydrated, and antigen retrieval was performed. Slides were immersed then permeabilized and blocked in 1% BSA in PBS. Tissues were then incubated overnight at 4 °C with primary antibody (Cx43 1:1000 (Sigma, C6219, St. Louis, MO, USA), αSMA 1:500 (Abcam, AB5694, Cambridge, UK), TGF-β1 1:400 (Abcam, AB215715)). No primary controls were included. Tissues were washed twice in washing buffer (0.05% PBS/Tween-20) and incubated with appropriate secondary antibody (Goat anti-rabbit AF488 1:500 (Thermo Fisher Scientific, A11008), Goat anti-rabbit AF555 1:500 (Thermo Fisher Scientific, A21422)) for 1 h. Tissues were washed twice and counterstained with DAPI (Life Technologies, Carlsbad, CA, USA, 1:10,000) and mounted with Citiflour^TM^ AF1 (Electron Microscopy Sciences, London, UK) mounting medium.

### 2.7. Picro-Sirius Red (PSR) Staining

Paraffin sections were rehydrated as previously described [35]. Sections were then stained with Picro-Sirius red (Abcam, UK) according to the manufacturer’s instructions and mounted using Organo mounting medium (Sigma, USA).

### 2.8. Brightfield and Confocal Microscopy

H&E and PSR slides were scanned on AxioScan.Z1 slide scanner (Zeiss, Gina, Germany). Immunofluorescence slides were imaged using a confocal microscope TCS SP8 (Leica, Wetzlar, Germany). Fluorophores were excited sequentially using a 405 nm, 488 nm and 532 nm wavelength laser. The images were 8-bit 1024 × 1024 pixels. All images were captured with identical parameters.

### 2.9. Microscopic Assessment–Inclusion and Exclusion Criteria

Tissue samples were subjected to inclusion/exclusion criteria to identify samples with features that allowed fair comparison. Only tissue sections with visible buttons or buttons with sutures only at the base of the button were selected. The exclusion criterion rejected tissue sections with no visible button or with a visible button accompanied by sutures at other locations other than at the base of the button (Figure S1B).

### 2.10. Extent of Inflammation

To score the extent of inflammation, each IB sample was divided into two regions, “Within IB” (WIB) and “Outside IB” (OIB) (Figure S1C). WIB region was defined as the area spanning 500 μm away from the periphery of the button while the OIB region was defined as the area encompassing 1500 μm away from both the left and right flanks of OIB. In each region, seven 62,500 μm^2^ region-of-interests (ROIs) were evenly and objectively distributed along the mesothelial edge of the tissue. Each ROI covers tissue containing both the mesothelium and the underlying muscle layer. Three blinded observers scored the extent of inflammation on a 5-point scale based on a scoring matrix described in Figure S1D.

### 2.11. Fibrosis

Several regions of the IB sample were evaluated. The entire fibrotic (EF) region was identified as the overall region that surrounds the immediate fibrotic (IF) region and other regions of adhesion-inducing determinants, such as IB and suture (IBS) (Figure S2A). The EF region was defined by the area occupied by abnormal ECM across the entire IB tissue and was surrounded at the base by thickened mesothelium. The IB could be defined as a “ball of muscle” and was distinguishable from the ECM. Suture material regions were determined by the presence of suture material (greyish brown). IF region was measured by subtracting the area of the IBS region from the calculated area of EF. Two ROIs were drawn on tissue sections stained with H&E, EF and IBS regions. Areas of these regions were measured using image analysis software Zen Lite 2.3 (Zeiss, Germany) and used to calculate the area of the IF region by subtracting IBS from EF. It was considered that the size of the IBS region could influence the size of the IF region, so the calculated area of the IF region was then normalised to the area of the IBS region.

### 2.12. Fibrosis Severity

ROIs were drawn around IF regions stained with PSR using ImageJ analysis software (NIH) (Figure S2B). These ROIs were saved for later use. The amount of collagen deposition was measured using the greyscale threshold method described by Schipke et al., (2017) [36]. Manual threshold values (80 to 255) were set on a greyscale image corresponding to the green channel for collagen detection and an output mask was generated (Figure S2B). The corresponding ROIs were loaded onto respective output masks and the detected area was measured (Figure S2B) and normalised to the IBS area.

### 2.13. Protein Levels of αSMA, Cx43 and TGF-β1

For αSMA, ROIs were used to demarcate regions of tissue with its expression, excluding areas of blood vessels with the oversaturated signal. In-house quantification algorithms were applied using ImageJ analysis software (NIH). Manual threshold values (80 to 255) were kept constant to quantify αSMA levels in the area of interest. The detected area was then measured and normalised to the IBS area. For Cx43 and TGF-β1, five fields of view were acquired from regions within and away from IB with ROIs drawn around mesothelial and muscle regions. Manual threshold values of (100 to 255) were set constant for Cx43 and TGF-β1 expression levels. The measured Cx43 and TGF-β1 pixel area were also normalised to the pixel area within the ROI.

### 2.14. Statistical Analysis

All data are presented as mean value ± standard deviation. All statistical comparisons between vehicle and Cx43asODN treatment groups were made using a Mann–Whitney *U* test, unless stated otherwise. A two-way analysis of variance (ANOVA) followed by a post-hoc Tukey’s multiple comparisons test was used for analysing protein levels of Cx43 and TGF-β1 or extent of inflammation in WIB and OIB regions after 6 and 24 h post-surgery. All statistical comparisons were performed using GraphPad Prism version 6.0, (GraphPad^TM^ Software, San Diego, CA, USA).

## 3. Results

### 3.1. Adhesions Were Observed as Early as 6 h Post-Surgery

The IB model was used for studying the role of Cx43 in post-surgical peritoneal adhesion. Three windows of assessments covering early and late phases of adhesiogenesis were chosen at 6 and 24 h post-surgery (n = 5 each) and 7 days post-surgery (n = 9) to evaluate intervention efficacy. Peritoneal adhesions were detected as early as 6 h post-surgery. IBs with attached adhesive bands were scored as 15 ± 13.7%. These adhesions were immature, filmy, avascular and broke easily with gentle traction. At 24 h the adhesions scored two-fold more (30 ± 20.9%). These were slightly more mature, denser, and less filmy. However, they were avascular and broke with gentle traction. The 7-day adhesions were much more mature, denser, and stronger, and required blunt or sharp dissection to break. They were more opaque, vascularised and extensive, with a mean score of 55.56 ± 27.3% (Figure S2C). We also observed a difference in the growth of adhesions (*p* = 0.0596) in the cranial and caudal intra-abdominal regions. Adhesions in the caudal abdomen (65%) were mostly attached to the epididymal fat pad (Figure S2C). Since the location of the IB can influence the quantity of adhesion created, subsequent analyses were then performed on IBs in the caudal abdomen to ensure consistent groups.

### 3.2. Significant Upregulation of Cx43 in IB during Early Adhesion Formation

**Figure 1:**
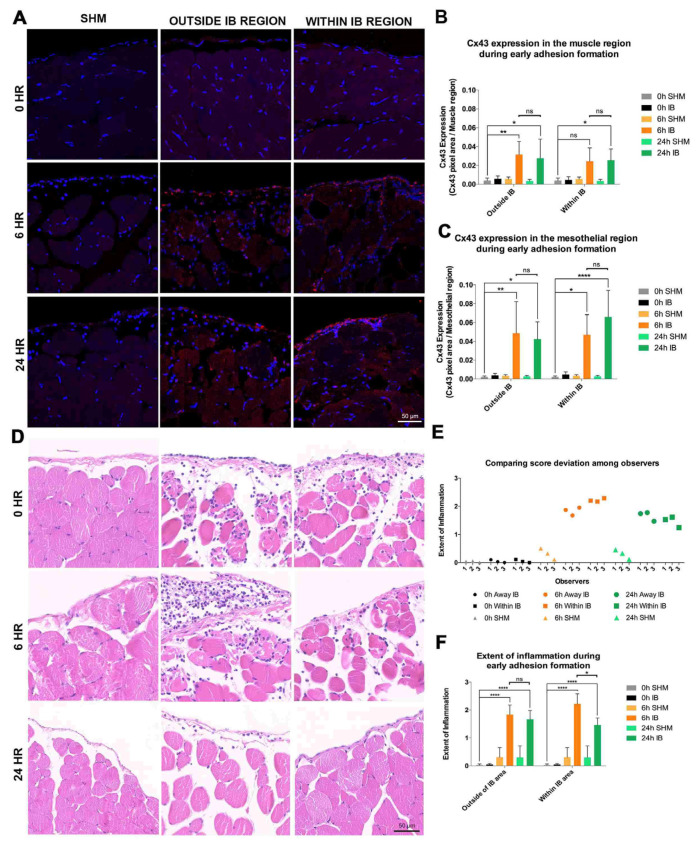
Cx43 was significantly upregulated at the IB regions during early adhesion formation. (**A**) Images of regions within and outside the IB samples at 0, 6, and 24 h post-surgery (n = 4, 5, 5). Longitudinal sections of IB sections Cx43 (red), and counterstained with DAPI (blue). IB samples at 0 h (top panels) revealed basal levels of Cx43 protein levels. At 6 h (middle panels), Cx43 levels were upregulated in both the mesothelial and muscle regions and were maintained for at least 24 h post-surgery (bottom panels). White dashed lines separate the mesothelial (upper) and muscle (lower) regions. Scale bar = 50 μm. (**B**) There were higher Cx43 levels at 6- and 24-h post-surgery. No changes in Cx43 levels in the muscle regions within the IB at 6 and 24 h post-surgery. (**C**) Significantly higher Cx43 levels in the mesothelial regions both within and outside the IB compared to 0 h sham-operated control (SHM). Data are presented as mean value ± standard deviation. Statistical comparisons were made using a two-way ANOVA followed by a post-hoc Tukey’s multiple comparisons test (* *p* ≤ 0.05, ** *p* ≤ 0.01, **** *p* ≤ 0.0001). (**D**) Inflammation significantly increased during early adhesion formation. H&E-stained IB samples at 0, 6 and 24 h post-surgery (n = 4, 5, 5). At 6 h post-surgery outside the IB region, moderate recruitment of leukocytes was observed. At 24 h post-surgery similar, but with fewer leukocytes in muscle further from the periphery. At 6 h post-surgery within the IB, more leukocytes were seen. Recruitment was reduced at 24 h. Scale bar = 50 μm. (**E**) Mean scores for each experimental group. (**F**) Inflammation within the IB at 6 h was significantly reduced at 24 h post-surgery. The inflammation in the IB at both 6 and 24 h was significant compared to 0 h sham-operated controls (SHM). Data are presented as mean value ± standard deviation. Statistical comparisons were made using a two-way ANOVA followed by a post-hoc Tukey’s multiple comparisons test (* *p* ≤ 0.05, **** *p* ≤ 0.0001).

Cx43 protein levels were investigated at 0, 6, and 24 h post-surgery (Figure 1A and Figure S3. Cx43 protein levels in sham-operated (SHM) mice provided reference levels. Image analysis was performed separately in muscle and mesothelial regions. At 6 h in the muscle WIB and WIB regions, the Cx43 protein level increased 6.3 and 8.1 fold (*p* ≤ 0.01) respectively (Figure 1B). A more dramatic increase in Cx43 was observed in the mesothelial regions with a 21-fold increase within (*p* ≤ 0.05) and outside IB (*p* ≤ 0.01) (Figure 1C). At 24 h Cx43 protein level remained significantly higher (*p* ≤ 0.05). No change in Cx43 protein level was observed in the muscle WIB region, but there was a further increase of 1.4-fold in the mesothelial WIB region (*p* ≤ 0.0001).

### 3.3. Increased Inflammation within IB during Early Adhesion Formation

Tissue samples from 0, 6, and 24 h post-surgery were stained with hematoxylin and eosin (H&E) (Figure 1D,E). At 6 h, moderate recruitment of leukocytes was detected within the muscle layer proximal to the periphery and mesothelial OIB regions (1.83 ± 0.34), whereas a larger recruitment of leukocytes was observed in WIB (2.21 ± 0.36) (Figure 1F). At 24 h, leukocytes recruited to OIB regions were reduced (1.66 ± 0.32) with a significant decrease in WIB (1.46 ± 0.25) (*p* ≤ 0.05) (Figure 1F). Inflammation in the IB at 6 and 24 h post-surgery was significantly higher than the basal state (0.05 ± 0.03) (*p* ≤ 0.0001) (Figure 1F).

### 3.4. Treatment with Cx43asODN Significantly Reduced Elevation of Cx43 in the Mesothelial WIB Region

**Figure 2:**
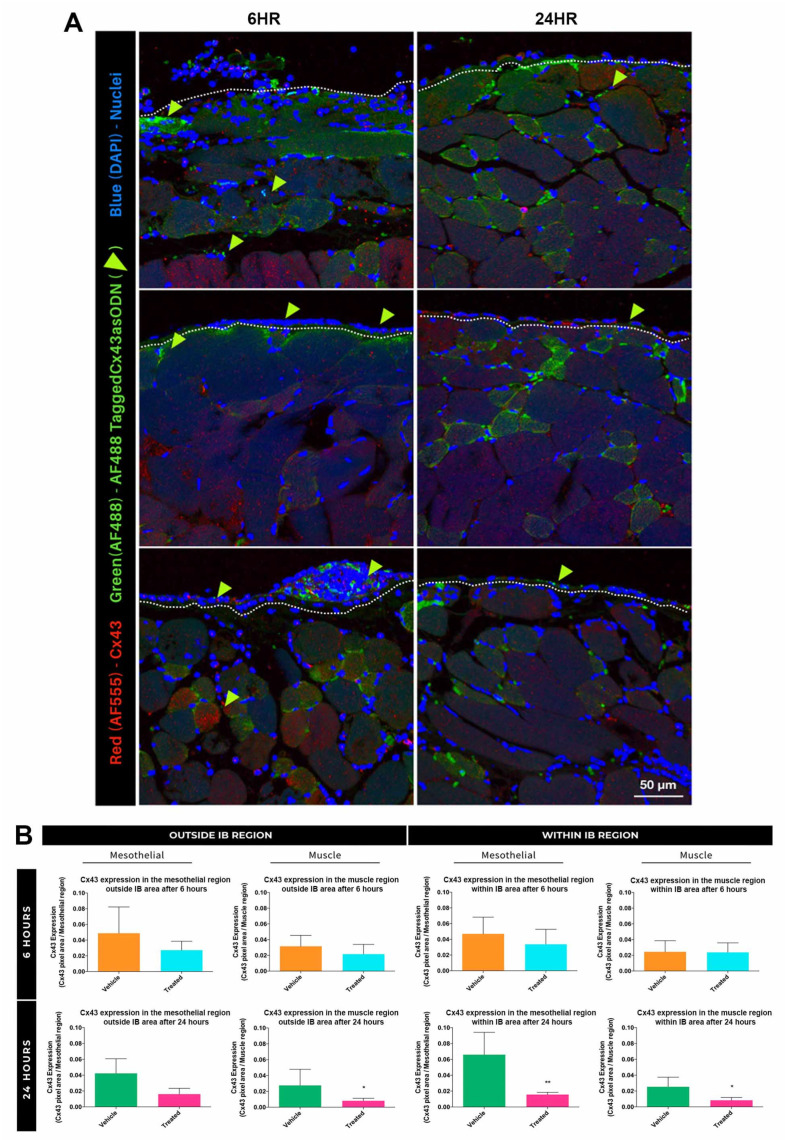
Cx43asODN significantly reduced Cx43 protein levels in the regions within the IB at 24 h post-surgery. (**A**) Images Cx43asODN-488 (green, with green arrowheads) and Cx43 protein (red), within the treated IBs at 6 and 24 h post-surgery (n = 3). At 6 h post-surgery Cx43asODN-488 was in the mesothelial regions and at least 180 μm into the muscle layer. At 24 h post-surgery Cx43asODN-488 was mostly detected proximal to the mesothelial edge. Cx43 protein levels in these treated samples were lower at 24 h post-surgery. White dashed lines separate the mesothelial (upper) and muscle (lower) regions. Scale bar = 50 μm. (**B**) significantly lower Cx43 protein levels in the mesothelial and muscle regions within the IB in the Cx43asODN-treated group (n = 6) compared to the vehicle-treated group (n = 5). Data are presented as mean value ± standard deviation. Statistical comparisons were made using a Mann-Whitney *U* test. (* *p* ≤ 0.05, ** *p* ≤ 0.01).

The majority of the Cx43asODN-488 signal was within the mesothelial layer of the IB (Figure 2A) and in the peripheral muscle penetrating at least 180 μm in 6 h. Cx43 protein levels were lower at 24 h. At 6 h, there was a slight decrease in Cx43 in the muscle and mesothelia within and outside IB (Figure 2B) and a prominent decrease at 24 h. In muscles within and away from IB, there was at least a 3-fold decrease in Cx43 (*p* ≤ 0.05). In mesothelia away from the IB, a 2.6-fold decrease was observed (*p* = 0.0519). The most significant decrease was 4.2 fold (*p* ≤ 0.01) in the mesothelia WIB.

### 3.5. Treatment with Cx43asODN Significantly Reduced Adhesion Strength and Severity at 7 Days Post-Surgery

**Figure 3:**
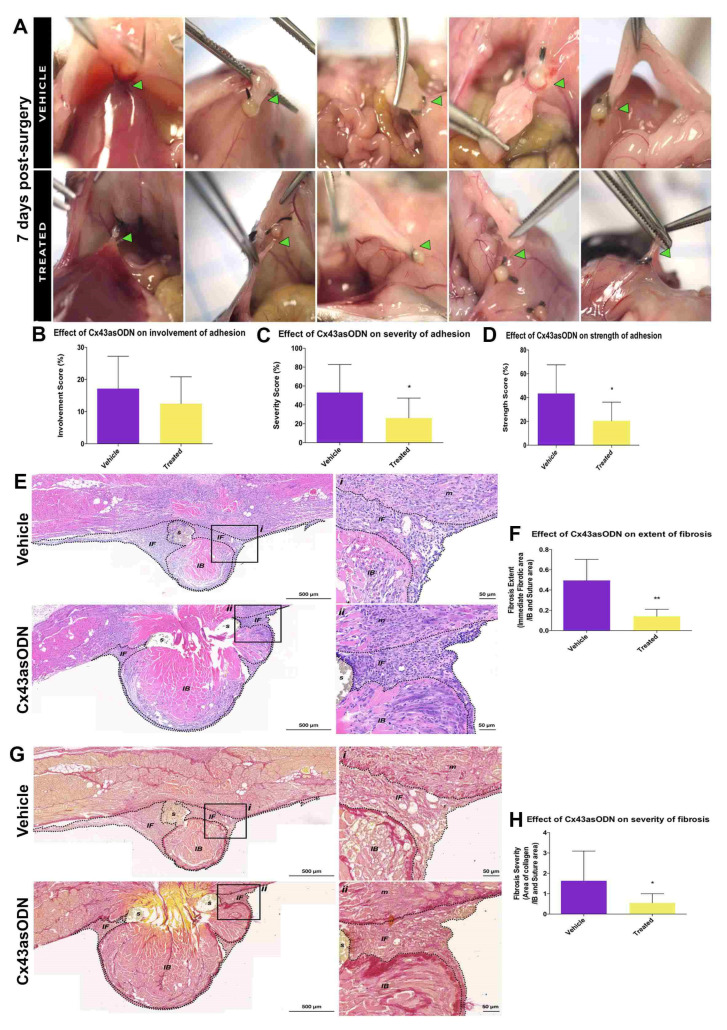
Treatment with Cx43asODN significantly reduced the severity and strength of adhesions. (**A**) Peritoneal adhesions in the lower abdomen at 7 days post-surgery. These adhesions (green arrowheads) were assessed for (**B**) involvement, (**C**) severity, and (**D**) strength, Cx43asODN (n = 10) and vehicle- (n = 9) treated groups. The Cx43asODN-treated groups had significantly lower severity and strength scores. Data are presented as mean value ± standard deviation. Statistical comparisons were made using a Mann–Whitney *U* test (* *p* ≤ 0.05). Treatment with Cx43asODN significantly reduced fibrosis of the adhesions. (**E**) H&E-stained IB samples of the vehicle- (n = 7) and Cx43asODN-treated (n = 5) groups at post-surgery day 7, and magnified regions. Scale bars 50 and 500 μm. (**F**) The extent of fibrosis of the adhesions in the Cx43asODN-treated group was significantly reduced compared to the vehicle-treated group. Data are presented as mean value ± standard deviation. Statistical comparisons were made using a Mann–Whitney *U* test (** *p* ≤ 0.01). Dashed lines highlight the boundary of the immediate fibrotic region. IB—ischaemic button; s—suture; m—muscle; IF—immediate fibrotic region. (**G**) PSR-stained IB samples of the vehicle- (n = 7) and Cx43asODN-treated (n = 5) groups at post-surgery day 7, and magnified regions (i, ii). Scale bars 50 and 500 μm. (**H**) The fibrotic severity of the adhesions of the Cx43asODN-treated group was significantly reduced compared to the vehicle-treated group. Data are presented as mean value ± standard deviation. Statistical comparisons were made using a Mann–Whitney *U* test (* *p* ≤ 0.05). Dashed lines highlight the boundary of the immediate fibrotic region. IB—ischaemic button; s—suture; m—muscle; IF—immediate fibrotic region.

While the significant reduction in Cx43 levels within the IB region at 24 h did not affect adhesion involvement (*p* = 0.295), the treatment with Cx43asODN significantly reduced adhesion severity (*p* ≤ 0.05) and strength at 7 days post-surgery (*p* ≤ 0.05) (Figure 3A,B). The adhesions in the vehicle-only group (53.0 ± 29.7) were opaque and dense compared to the treatment group (26.1 ± 21.1) (Figure 3C) The adhesions in the vehicle-only group required blunt dissection to break (43.5 ± 24.0) whereas adhesions in the treated group required little effort to break (20.0 ± 15.7) (Figure 3D).

### 3.6. Cx43asODN Treatment Significantly Reduced Fibrosis and Severity of Adhesions

Fibrotic regions in stained&E-stained IB samples were measured. Fibrosis in Cx43asODN-treated group (0.14 ± 0.07) was significantly reduced compared to the vehicle only group (0.49 ± 0.2) (*p* ≤ 0.01) (Figure 3E,F). The amount of collagen within fibrotic regions of the Cx43asODN-treated group (0.55 ± 0.45) was significantly less than the vehicle-only group (1.63 ± 1.5) (*p* ≤ 0.05) (Figure 3G,H).

### 3.7. Effect of Cx43asODN Treatment on the Plasminogen Activating Activity

**Figure 4:**
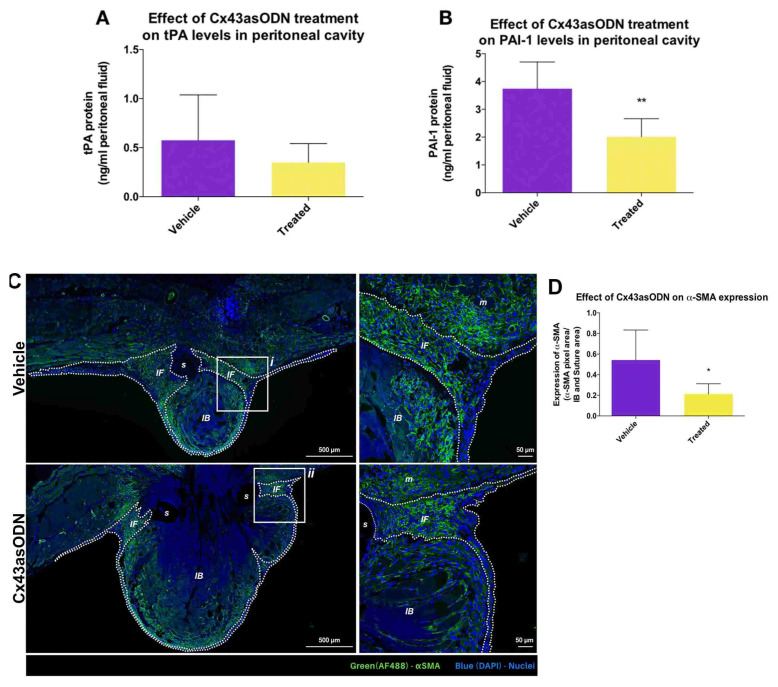
Effect of Cx43asODN treatment on tissue plasminogen activator (tPA) and plasminogen activator inhibitor-1 (PAI) proteins in the peritoneal fluid at day 7 post-surgery. (**A**) tPA protein levels at day 7 post-surgery were unchanged by Cx43asODN treatment (n = 7). (**B**) Treatment with Cx43asODN significantly reduced PAI-1 protein levels at day 7 post-surgery when compared to the vehicle-treated group. Statistical comparisons were made using a Mann–Whitney *U* test (** *p* ≤ 0.01). Treatment with Cx43asODN significantly reduced αSMA protein levels within the fibrotic region. (**C**) Images of IB of the vehicle- (n = 7) and Cx43asODN-treated (n = 5) groups at 7 days post-surgery, and magnified regions (i, ii). Scale bars 50 and 500 μm. αSMA (green), and counterstained with DAPI (blue). (**D**) Significantly reduced αSMA protein levels within the immediate fibrotic region in the Cx43asODN-treated group. Data are presented as mean value ± standard deviation. Statistical comparisons were made using a Mann–Whitney *U* test (* *p* ≤ 0.05). Dashed lines highlight the immediate fibrotic region. IB—ischaemic button; s—suture; m—muscle; IF—immediate fibrotic region.

The protein level of tPA and PAI-1 in the peritoneal cavity was investigated (Figure 4). Under pathological conditions, PAA is decreased, leading to the development of adhesions. Treatment with Cx43asODN did not affect the protein level of tPA in the peritoneal fluid (*p* = 0.263) (Figure 4A). In contrast, the protein level of PAI-1 was significantly lower (*p* ≤ 0.01) (Figure 4B) suggesting higher PAA activity in the Cx43asODN-treated group.

### 3.8. Treatment with Cx43asODN Significantly Reduced Fibroblast Activation

The αSMA staining was mostly in the regions proximal to the suture regions of IB (Figure 4C). In immediate fibrotic regions, its expression was significantly lower in the Cx43asODN-treated group (*p* ≤ 0.05) (Figure 4D) suggesting a lower degree of fibroblast activation.

### 3.9. Treatment with Cx43asODN Reduced the Extent of Adhesion Formation

**Figure 5:**
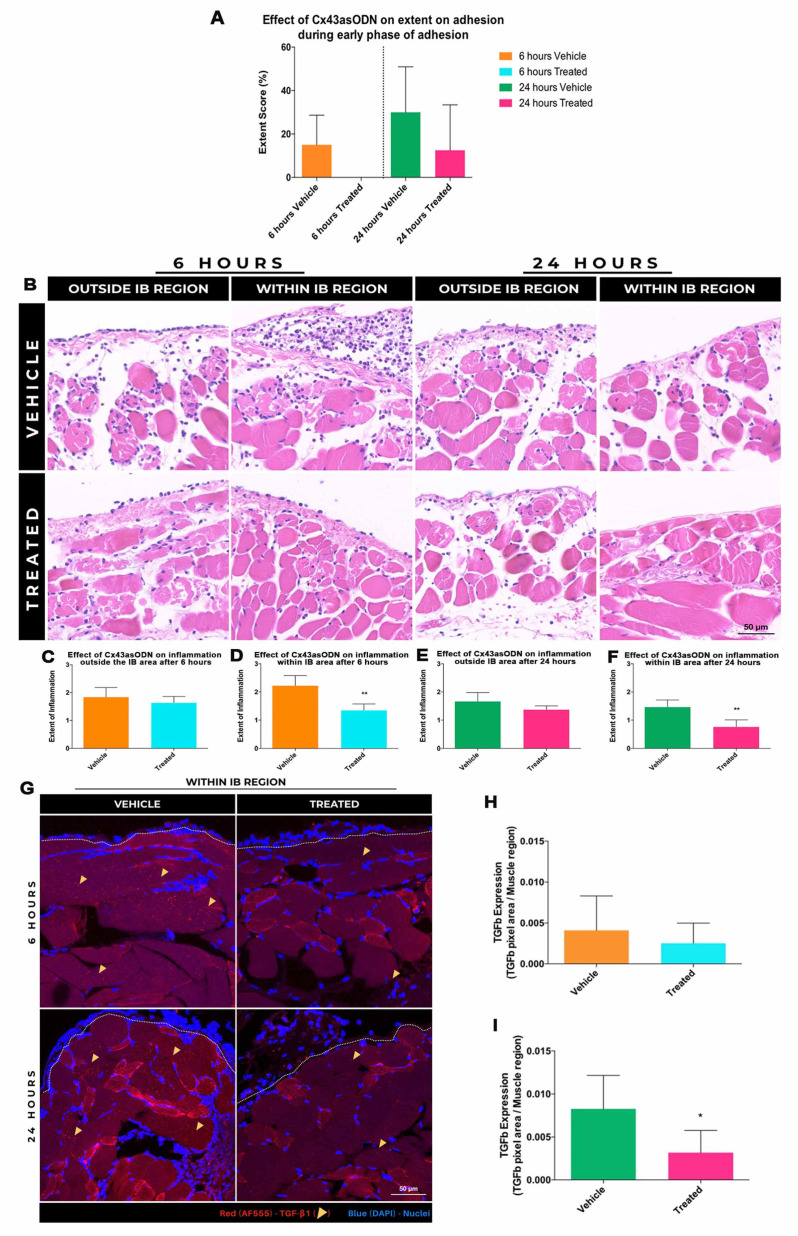
Cx43asODN treatment reduced the extent of adhesions during early adhesion formation. (**A**) Peritoneal adhesions in the abdomen were scored at 6 and 24 h post-surgery vehicle- (n = 5) and Cx43asODN-treated (n = 6) groups. At 6 h, the vehicle-treated group had a score of 15% while no adhesions were observed in the Cx43asODN-treated group. At 24 h, the vehicle-treated group had a score of 30% while the Cx43asODN-treated group had about two fold less. Data is presented as mean value ± standard deviation. Statistical comparisons between the treated groups and their respective time-matched control were made using a Mann-Whitney *U* test. Cx43 asODN treatment significantly reduced inflammation within the IB at 6 and 24 h. (**B**) Representative ROIs of H&E-stained IB samples at 6 and 24 h post-surgery. Outside of the IB after 6 and 24 h, no changes in inflammation were observed although there was a reduction within the IB of the Cx43 asODN-treated group at both time points. Scale bar = 50 μm. No differences were found between the vehicle-treated (n = 5) and Cx43 asODN-treated group (n = 6) in the regions outside of IB after 6 (**C**) and 24 h (**D**). A significant decrease in inflammation within the IB was found in the treated-groups after 6 h (**E**) and 24 h (**F**). Data is presented as mean value ± standard deviation. Statistical comparisons were made using a Mann-Whitney *U* test (** *p* ≤ 0.01). Treatment with Cx43asODN reduced TGF-β1 protein levels within the IB during early phase adhesion formation. (**G**) Images of TGF-β1 (red, highlighted with yellow arrowheads), within the IB after 6 and 24 h post-surgery. TGF-β1 protein in the muscle in the vehicle treated-group (n = 5) after (**H**) 6- and (**I**) 24-h were slightly reduced in the Cx43asODN treated group (n = 6). White dashed lines separate the mesothelial (upper) and muscle (lower) regions. Scale bar = 50 μm. Data is presented as mean value ± standard deviation. Statistical comparisons were made using a Mann-Whitney *U* test. (* *p* ≤ 0.05).

The effect of Cx43asODN treatment on early adhesion formation was macroscopically evaluated [32] at 6 and 24 h post-surgery (Figure 5A). At 6 h, the mean extent of adhesion in the vehicle-only group was 15 ± 13.7%. In contrast, no peritoneal adhesions were detected in the Cx43asODN-treated group (*p* = 0.06). At 24 h post-surgery, the mean extent of adhesions in the treated group (12.5 ± 20.9%) was slightly reduced compared to the vehicle-only group (30 ± 20.9%) (*p* = 0.275).

### 3.10. Effect of Cx43asODN Treatment on Inflammation during Early Adhesion Formation

At both 6 and 24 h post-surgery, Cx43asODN treatment showed a mild reduction in inflammation OIB (*p* = 0.528, *p* = 0.222, respectively) and a more significant reduction WIB when compared to control (*p* ≤ 0.01) (Figure 5). Staining for TGF-β1 was elevated in the muscle WIB (Figure 5G). Lower levels of TGF-β1 in the immediate fibrotic regions were observed in Cx43asODN treated group when compared to vehicle-only group at 6 (*p* = 0.424) and 24 h (*p* = 0.31) (Figure 5H,I).

## 4. Discussion

Our IB model was able to generate adhesions consistent with the clinical Type 1B definition. This is classified as “de novo adhesions that are produced at sites where surgical procedures were performed, previously clear of adhesions” [37]. Thickening of mesothelium was observed in our IB model [38] and this was consistent with patient reports [28,39,40]. Evaluation of adhesion formation in other versions of this model has been previously performed at time points from 12 h to 24 weeks [41]. Here, some adhesions were detected as early as 6 h, although they were infrequent. Early adhesions were immature in morphology and could be broken with gentle traction. The extent of adhesions doubled over 24 h despite immaturity, transparency, filmy, avascular and could be broken with little effort. In contrast, adhesions at 7 days were opaque, dense, vascular, and required definitive blunt dissection to break. The extent was two-fold more than 24-h post-surgery. Two-thirds of 7-day adhesions were found in the epididymal fat pad of the caudal abdomen (Figure S2). Earlier studies revealed fibrinolytic activity among tissues in humans and animals to be heterogenous [42]. Almost four times more fibrinolytic activity was documented from surgical biopsies of serosal tissues from omentum compared to gall bladder [43]. However, the understanding of the fibrinolytic activity of tissues under physiological and pathological conditions is still limited.

During early adhesion formation, an elevated Cx43 protein level was observed (Figure S2). The most significant increase of Cx43 was at 24 h. Based on the position and morphology of Cx43 +VE cells coupled with H&E sister sections, we speculate that increased Cx43 was mainly in the mesothelial cells and infiltrating leukocytes (Figure 5). Cx43 is a major gap junction in human pleural [44] and peritoneal mesothelial cells [45,46,47]. Previous studies of Cx43 in these cell types mainly investigated its involvement in cancer and not in adhesion formation [45,46,47]. An increase in Cx43 protein in neutrophils and monocytes/macrophages during inflammation has been documented [48,49,50,51]. Increased levels of Cx40 and Cx43 were seen in neutrophils stimulated with TNF-α in endothelial cell-conditioned media [51]. ATP can also be released from Cx43 hemichannels in neutrophils and monocytes under inflammatory conditions [52]. ATP can activate purinergic receptors on endothelial cells, triggering a propagation of Ca^2+-^dependent signalling across the endothelium, which results in the expression of leukocyte adhesion molecules such as P-selectin [53]. This may facilitate the recruitment of leukocytes during early adhesion pathogenesis. Here, we found that reducing the Cx43 with Cx43asODN in IB significantly reduced leukocyte recruitment and microabscesses.

Following peritoneal injury, the release of cytokines and chemokines triggers a cascade of proinflammatory events during the healing process. Early responder cells are predominantly neutrophils, with a gradual shift to macrophages [54]. The recruited leukocytes initiate another cytokine cascade, which is speculated to be crucial for adhesion formation [54,55]. This was demonstrated by cyclophosphamide-mediated reduction in neutrophils led to a reduced number and severity of adhesions [56]. In another study, disrupting the interaction between chemokine ligand 1 and its receptor, chemokine receptor 8 in peritoneal macrophages hampers their migration, resulting in a significant reduction of peritoneal adhesions [57]. Here there was a significant reduction in leukocytes within the IB region at 6 and 24 h in the Cx43asODN treated group. In other disease models such as acute lung injury from lipopolysaccharide, almost 50% fewer neutrophils were found in bronchoalveolar lavages of Cx43^+/−^ mice or mice treated with connexin mimetic peptide, Gap26 [58]. Similarly, in hypertension-induced and tubulointerstitial inflammation models of chronic kidney disease, the infiltration of monocytes was significantly reduced with decreased Cx43 mRNA expression in Cx43^+/−^ mice or when treated with Cx43asODN [26]. Although these observations were made in different disease models, these findings corroborate the emerging role of Cx43 in leukocyte recruitment. Future investigations using in vitro systems or conditional Cx43 transgenic mice will help determine which cell-derived Cx43 is a stronger contributor to leukocyte recruitment. This may elucidate a method to fine-tune the recruitment of leukocytes to the site of injury, presenting a potential therapeutic avenue. Moreover, the treatment of lymphocytes with connexin mimetic peptides, Gap26 and Gap27, inhibited the synthesis of cytokines [59]. Although no appreciable changes in cytokine levels were observed at 6 h post-surgery, TGF-β1 levels were significantly reduced in the muscle regions within the IB at 24 h post-surgery in the Cx43asODN-treated group.

The differentiation of fibroblasts into myofibroblasts in response to profibrotic cytokines is characterized by the expression of αSMA [60]. Proposed to be a profibrotic mediator [61], TGF-β1 has been shown to promote the expression of αSMA, regulating the myofibroblast conversion [21,60,62,63]. Work by Dai and colleagues demonstrated Cx43 positively mediates the activity of TGF-β through competitive binding to microtubules between endogenous Smads and Cx43 [22]. Later work by the same group demonstrated that over-expression or inhibition of Cx43 respectively potentiated or inhibited TGF-β1-induced myofibroblast conversion [21].

The presence of TGF-β1 in muscle injury has been well documented [64,65,66]. In skeletal muscle, TGF-β1 is regarded as one of the most profibrogenic factors [67], capable of regulating synthesis and degradation and remodelling of the ECM [65,67]. Upregulation of TGF-β1 and accompanying fibrosis were observed in skeletal muscles in response to damage and denervation [68]. Treatment of TGF-β1 and TβR1 with antibodies to impair TGF-β1 activity leads to marked improvements in the regeneration of muscle with reduced fibrosis [68]. In this study, TGF-β1 protein level at 24 h post-surgery was significantly reduced in the muscle region within the IB in the Cx43asODN treated group (Figure 5). Although fibrosis in the muscle within the IB was not investigated, αSMA protein levels were lower in this region in the Cx43asODN treated group. It is interesting to note that a single treatment with the Cx43asODN at the start had ongoing effects on the course of adhesion biology and lessened their long-term development.

The formation of peritoneal adhesions depends on the equilibrium between fibrin production and fibrinolysis following peritoneal injury [28]. The complete degradation of fibrin facilitates normal peritoneal healing. However, incomplete degradation of fibrin may serve as a matrix for invading fibroblasts and the growth of new blood vessels in adhesion formation [28]. Plasminogen activators play a vital role in the fibrinolytic sequence. They convert inactive plasminogen substrates to an active form, plasmin, which then dissolves fibrin. In the peritoneal cavity, 95% of the plasminogen-activating activity (PAA) is contributed by tPA [69]. However, the activity of plasminogen activators also depends on the presence of PAIs. PAI-1, the main fibrinolytic inhibitor, hampers the fibrinolytic response by antagonising and forming inactive complexes with tPA [54]. In this study, protein levels of PAI-1 in the peritoneal fluid of the Cx43asODN-treated group was significantly lower than the vehicle-treated group suggesting PAA activity will be higher at day 7 post-surgery Reduced levels of PAA were suggested to increase fibrin deposition, which facilitates invasion of fibroblasts and production of extracellular matrix, leading to the development of adhesions [28,29].

Work investigating the association between Cx43 and tPA levels is limited. Earlier in vivo studies on the vascular effects of rotigaptide, a synthetic peptide capable of potentiating Cx43 communication, revealed that Cx43 communication has no effect on tPA release in the forearm arterial circulation [70]. In this study, the treatment with Cx43asODN did not appear to affect the protein level of tPA in the peritoneal fluid. While these observations may suggest little association between Cx43 and tPA, smooth muscle function inhibitor, atorvastatin-treated human vascular smooth muscle cells were observed to decrease Cx43 protein levels, which was accompanied by an increase of mRNA expression of tPA. This suggests the effect of Cx43 on tPA expression may be cell-type dependent [71]. In the same study, the decrease in Cx43 protein level was accompanied by a decrease of PAI-1 protein. Reduction of Cx43 activity, via nonspecific gap junction inhibitors (carbenoxolone and probenecid) or siRNA knockdown, decreased PAI-1 mRNA expression in rat cardiac fibroblasts, inhibiting the ATP release involved in profibrotic signaling [72]. The decrease in ATP levels was accompanied by a decrease in αSMA protein and collagen accumulation [72]. Overall, this may suggest that the Cx43-mediated reduction of PAI-1 expression is a result of basal nucleotide signalling and may help explain the significant reduction of PAI-1 levels in the Cx43asODN-treated group. Collectively, the early intervention with a single treatment with Cx43asODN has the potential to reduce the TGF-β1 signalling pathway to alter α-SMA expression and PAI-1 levels, which in turn reduces profibrotic signalling and adhesion formation.

## 5. Conclusions

We set out to explore the role of Cx43 in an ischaemic button model of adhesion formation. During early adhesion formation increase of Cx43 protein accompanied by leukocyte recruitment was seen in the mesothelial regions of the ischaemic button at 6 and 24 h post-surgery. Cx43asODN, significantly suppressed the elevation of Cx43 protein levels and adhesion strength and severity, with reduced fibrosis and αSMA protein levels. Collectively, this suggests that the elevation of Cx43 protein levels plays an important role in adhesion formation and may be a potential therapeutic target for the prevention of post-surgical peritoneal adhesions.

## Data Availability

Not applicable.

## Supplementary Materials

The following supporting information can be downloaded at: https://www.mdpi.com/article/10.3390/life12111734/s1. Figure S1. A Ischemic button formation in mice. Disinfection of the incision site after shaving. Midline incision of the skin. Two IBs, spaced approximately 1 cm apart, were created on the peritoneum on each side of the midline incision. Wound closure by applying simple interrupted sutures to the peritoneal musculature and metallic clips to the skin. B Inclusion and exclusion criteria. The inclusion criterion included only tissues sections with a visible button or with a visible button accompanied with sutures only at the base of the button. The exclusion criterion rejected tissues sections with no visible button or with a visible button accompanied with sutures found at other locations other than at the base of the button. IB–Ischaemic Button region, S–Suture region. C Designating ROIs for scoring the extent of inflammation. Seven ROIs (black squares) were taken each from “Within IB” and “Outside IB” regions. The “Within IB” region was defined as the area spanning 500 μm away from the periphery of the button (green box). The “Outside IB” region was defined as the area encompassing 1500 μm away from both left and right flanks of the perimeter of the “Within IB” region (blue box). IB–Ischaemic Button region, S–Suture region. D Scoring matrix for grading extent of inflammation. Regions-of-interests were graded for the extent of inflammation based the above scoring matrix. Three representative images accompanied the description of each grade before blinded assessment. Grade 0, little/no recruitment of leukocytes to the edge or muscle area; Grade 1, some recruitment of leukocytes to the edge but little/none to the muscle area; Grade 2, moderate recruitment of leukocytes to the edge and/or to the muscle area (no focal accumulation of leukocytes); Grade 3, large recruitment of leukocytes to the edge and/or to muscle area (some focal accumulation of leukocytes); Grade 4, extensive recruitment of leukocytes, which is limited to a particular region; Figure S2. A Defining regions around the IB. Representative image of a section of the IB sample stained with H&E. The entire fibrotic (EF) region was identified as the total fibrotic region (red dashed lines). This is made up of the immediate fibrotic (IF) region and the regions of adhesion-inducing determinants such as the IB and suture (IBS) (black dashed lines). The immediate fibrotic region (IF) determined by drawing ROIs to exclude the FR region from the IBS region or by the subtracting the FR region from IBS region. B–Ischaemic Button region, S–Suture region. B Example of image analysis workflow for PSR stained images. ROIs were drawn around the IF regions of tissue sections stained with PSR using ImageJ. The images were converted to the RGB stacks, which generates greyscale images for red, green, and blue channels. Manual threshold was performed on the greyscale image corresponding to the green channel to detect collagen, which would produce an output mask. Example of output mask after manual threshold (80 to 255). Specific threshold values were kept constant throughout analysis. Example of the loading of ROIs for the respective sections so that the measure tool can be used to detect the area of interest. C Types of adhesions observed at early and late phases of adhesion development. Images of adhesions (highlighted with green arrowheads) early phases (6 and 24 h, n = 5 each) and late phase (7 days (n = 9)). A filmy, avascular adhesion between the IB and the epididymal fat pad, at 6 h post-surgery. These were avascular and required gentle traction to break. At 24 h, a denser, less filmy, avascular adhesion between the IB and the mesenteric fat was detected. Extent of adhesions observed at early and late phase of adhesion development. Adhesions at 7 days were more mature. Examples of opaque and vascular adhesive bands requiring blunt dissection to break, between the IB and a liver lobe or a small bowel loop at 7 days. Examples of dense, opaque, and vascular adhesive bands requiring sharp dissection to break, detected between the IB and epididymal fat pad. Majority of the adhesions at 7 days were in the lower abdominal region. Fat pad distribution in the left abdominal region. Data is presented as mean value ± standard deviation. RP–Retroperitoneal fat pad; E–Epididymal fat pad; Figure S3. Schematic diagram of Cx43 protein levels during early adhesion formation. Relative protein levels of Cx43 in the mesothelial (circles) and muscle (squares) regions within and outside the IB are illustrated using shapes. The sizes of the shapes represent their fold change from basal physiological levels. Highest Cx43 protein levels were detected in the mesothelial regions within the IB at 24 h post-surgery. Statistically significant changes in Cx43 protein levels at 24 h post-surgery due to Cx43asODN treatment are denoted with downward pointing arrows: single arrow, (*p* ≤ 0.05); two arrows, (*p* ≤ 0.01). Cx43 protein level at 6 h was not significantly reduced with treatment of Cx43asODN; Table S1. Scoring matrix for assessing peritoneal adhesions.
