# Supplementary material for: Connexin43 in Post-Surgical Peritoneal Adhesion Formation
Source: Life (Basel). 2022 Oct 28;12(11):1734. doi: 10.3390/life12111734 (PMC9697983; doi:10.3390/life12111734)
Supplement: Supplementary file 1 [file life-12-01734-s001.zip › Cx43 adhesion Supplemental Figure Legend.pdf]

Title: Connexin43 in post-surgical peritoneal adhesion formation

Authors: Jia Wang Chua[1,2], Moogaambikai Thangaveloo[1,2], Debbie Lim[1,2], Leigh E. Madden[1,2], Anthony RJ. Philips[3] and David L. Becker[1,2]

1. Lee Kong Chian School of Medicine, Nanyang Technological University Singapore, Clinical Sciences Building, 11, Mandalay Road, Singapore, 308232
2. Skin Research Institute Singapore, Level 17, Clinical Sciences Building, 11, Mandalay Road, Singapore, 308232
3. School of Biological Sciences, Auckland University, Auckland, New Zealand

ORCID

JWC

MT

DL

SBHL

LM

ARJP 0000-0001-6143-5866

DLB 0000-0002-9300-0899

Correspondence to:

DLB Lee Kong Chian School of Medicine, Nanyang Technological University Singapore, Clinical Sciences Building, 11, Mandalay Road, Singapore, 308232

e-mail: David.becker@ntu.edu.sg

Key words: 4-6

Gap Junctions, Connexins, Inflammation, Fibrosis, Peritoneal adhesions

Supplemental Figure Legend

Figure S1. A Ischemic button formation in mice. Disinfection of the incision site after shaving. Midline incision of the skin. Two IBs, spaced approximately 1 cm apart, were created on the peritoneum on each side of the midline incision. Wound closure by applying simple interrupted sutures to the peritoneal musculature and metallic clips to the skin. **B** Inclusion and exclusion criteria. The inclusion criterion included only tissues sections with a visible button or with a visible button accompanied with sutures only at the base of the button. The exclusion criterion rejected tissues sections with no visible button or with a visible button accompanied with sutures found at other locations other than at the base of the button. *IB* – Ischaemic Button region, *S* – Suture region. **C** Designating ROIs for scoring the extent of inflammation. Seven ROIs (black squares) were taken each from “Within IB” and “Outside IB” regions. The “Within IB” region was defined as the area spanning 500 µm away from the periphery of the button (green box). The “Outside IB” region was defined as the area encompassing 1500 µm away from both left and right flanks of the perimeter of the “Within IB” region (blue box). *IB* – Ischaemic Button region, *S* – Suture region. **D** Scoring matrix for grading extent of inflammation. Regions-of-interests were graded for the extent of inflammation based the above scoring matrix. Three representative images accompanied the description of each grade before blinded assessment. Grade 0, little/no recruitment of leukocytes to the edge or muscle area; Grade 1, some recruitment of leukocytes to the edge but little/none to the muscle area; Grade 2, moderate recruitment of leukocytes to the edge and/or to the muscle area (no focal accumulation of leukocytes); Grade 3, large recruitment of leukocytes to the edge and/or to muscle area (some focal accumulation of leukocytes); Grade 4, extensive recruitment of leukocytes, which is limited to a particular region.

Figure S2. A Defining regions around the IB. Representative image of a section of the IB sample stained with H&E. The entire fibrotic (EF) region was identified as the total fibrotic region (red dashed lines). This is made up of the immediate fibrotic (IF) region and the regions of adhesion-inducing determinants such as the IB and suture (IBS) (black dashed lines). The immediate fibrotic region (IF) determined by drawing ROIs to exclude the FR region from the IBS region or by the subtracting the FR region from IBS region. *B* – Ischaemic Button region, *S* – Suture region. **B** Example of image analysis workflow for PSR stained images. ROIs were drawn around the IF regions of tissue sections stained with PSR using ImageJ. The images were converted to the RGB stacks, which generates greyscale images for red, green, and blue channels. Manual threshold was performed on the greyscale image corresponding to the green channel to detect collagen, which would produce an output mask. Example of output mask after manual threshold (80 to 255). Specific threshold values were kept constant throughout analysis. Example of the loading of ROIs for the respective sections so that the measure tool can be used to detect the area of interest. **C** Types of adhesions observed at early and late phases of adhesion development. Images of adhesions (highlighted with green arrowheads) early phases (6 and 24 hours, n=5 each) and late phase (7 days (n=9)). A filmy, avascular adhesion between the IB and the epididymal fat pad, at 6 hours post-surgery. These were avascular and required gentle traction to break. At 24 hours, a denser, less filmy, avascular adhesion between the IB and the mesenteric fat was detected. Extent of adhesions observed at early and late phase of adhesion development. Adhesions at 7 days were more mature. Examples of opaque and vascular adhesive bands requiring blunt dissection to break, between the IB and a liver lobe or a small bowel loop at 7 days. Examples of dense, opaque, and vascular adhesive bands requiring sharp dissection to break, detected between the IB and

epididymal fat pad. Majority of the adhesions at 7 days were in the lower abdominal region. Fat pad distribution in the left abdominal region. Data is presented as mean value ± standard deviation. *RP* –Retroperitoneal fat pad; *E* – Epididymal fat pad.

Figure S3. Schematic diagram of Cx43 protein levels during early adhesion formation. Relative protein levels of Cx43 in the mesothelial (circles) and muscle (squares) regions within and outside the IB are illustrated using shapes. The sizes of the shapes represent their fold change from basal physiological levels. Highest Cx43 protein levels were detected in the mesothelial regions within the IB at 24 hours post-surgery. Statistically significant changes in Cx43 protein levels at 24 hours post-surgery due to Cx43asODN treatment are denoted with downward pointing arrows: single arrow, ($p \leq 0.05$); two arrows, ($p \leq 0.01$). Cx43 protein level at 6 hours was not significantly reduced with treatment of Cx43asODN.
