# Supplementary material for: Connexin43 in Post-Surgical Peritoneal Adhesion Formation
Source: Life (Basel). 2022 Oct 28;12(11):1734. doi: 10.3390/life12111734 (PMC9697983; doi:10.3390/life12111734)
Supplement: Supplementary file 1 [file life-12-01734-s001.zip › Table S1.pdf]

Table S1. Scoring matrix for assessing peritoneal adhesions

Variable	Score	Description
**Extent [35]**	0	Complete absence of adhesions
	1	Only 1 button has an adhesion band
	2	2 buttons have adhesion bands
	3	3 buttons have adhesion bands
	4	4 buttons have adhesion bands
**Involvement (Quantity) [37]**	0	Complete absence of adhesions
	1	1 adhesion band between the organs or between one organ and abdominal wall
	2	2 adhesion bands between the organs or between one organ and abdominal wall
	3	>2 adhesion bands between the organs or between one organ and abdominal wall or adhesions of intestinal loops without any adhesion to the abdominal wall. Significant adhesion.
	4	Adhesion of all viscera to the abdominal wall
**Severity (Quality) [34]**	0	Complete absence of adhesions
	1	Filmy, avascular
	2	Dense, opaque and/or vascular
	3	Very thick cohesive attachment
**Strength [36]**	0	Complete absence of adhesions
	1	Gentle traction requiring little effort to break adhesion
	2	Blunt dissection requiring a blunt instrument to break adhesion
	3	Sharp dissection requiring a sharp instrument to break adhesion
	4	Adhesion of all viscera to the abdominal wall
