# Supplementary figures and images for: Connexin43 in Post-Surgical Peritoneal Adhesion Formation
Source: Life (Basel). 2022 Oct 28;12(11):1734. doi: 10.3390/life12111734 (PMC9697983; doi:10.3390/life12111734)

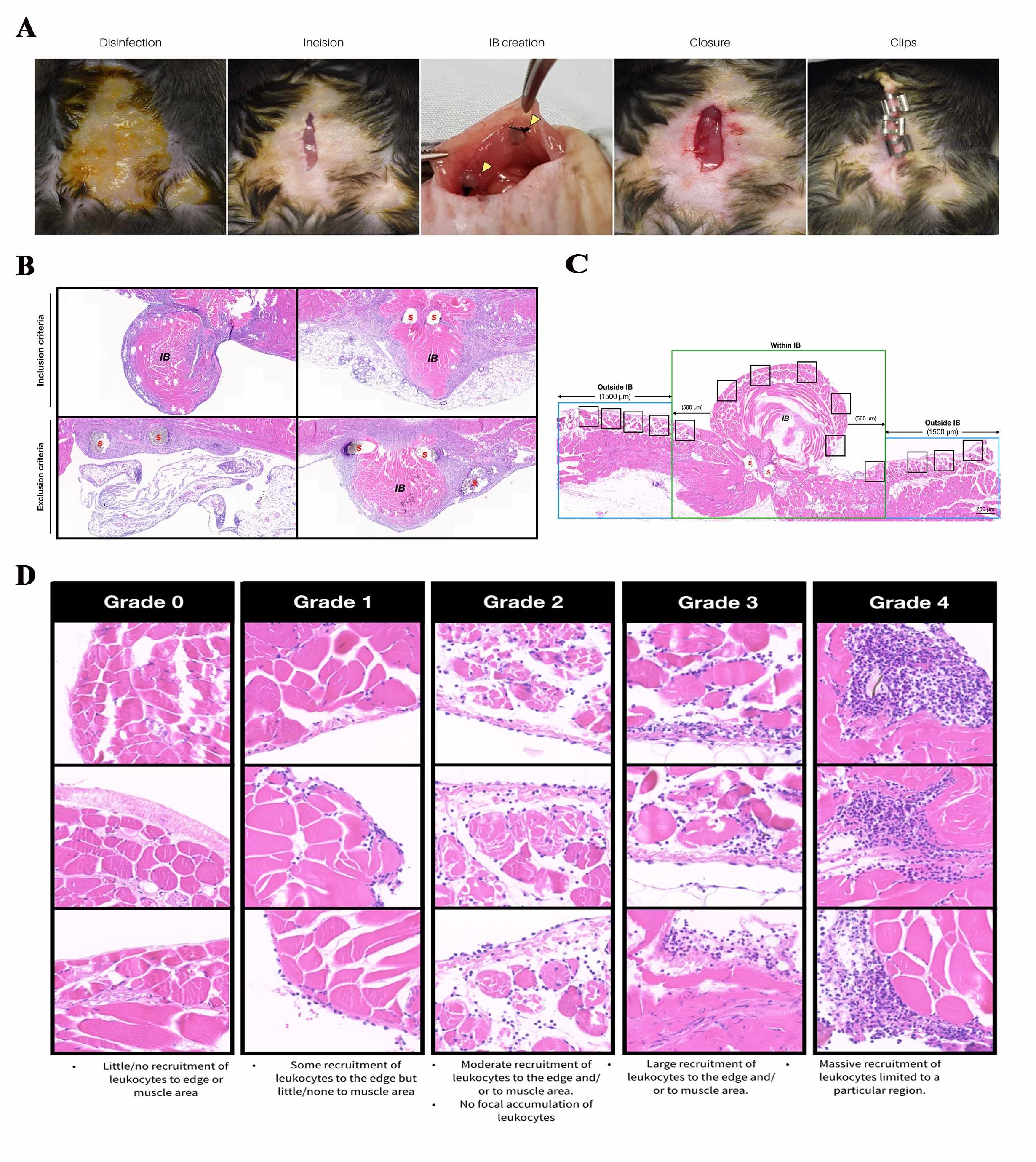

Supplement: Supplementary file 1 [file life-12-01734-s001.zip › Figure S1.jpg]

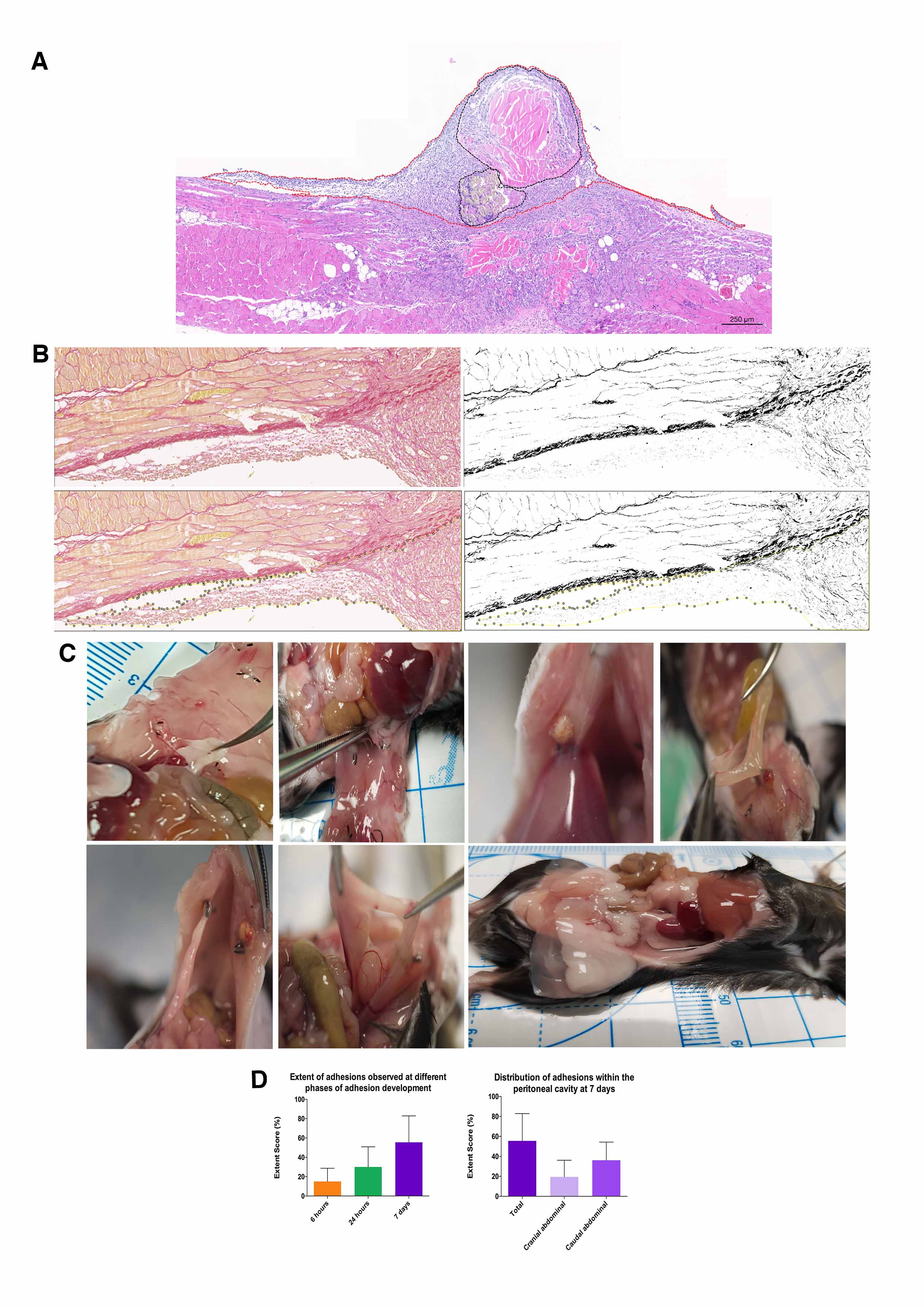

Supplement: Supplementary file 1 [file life-12-01734-s001.zip › Figure S2.jpg]

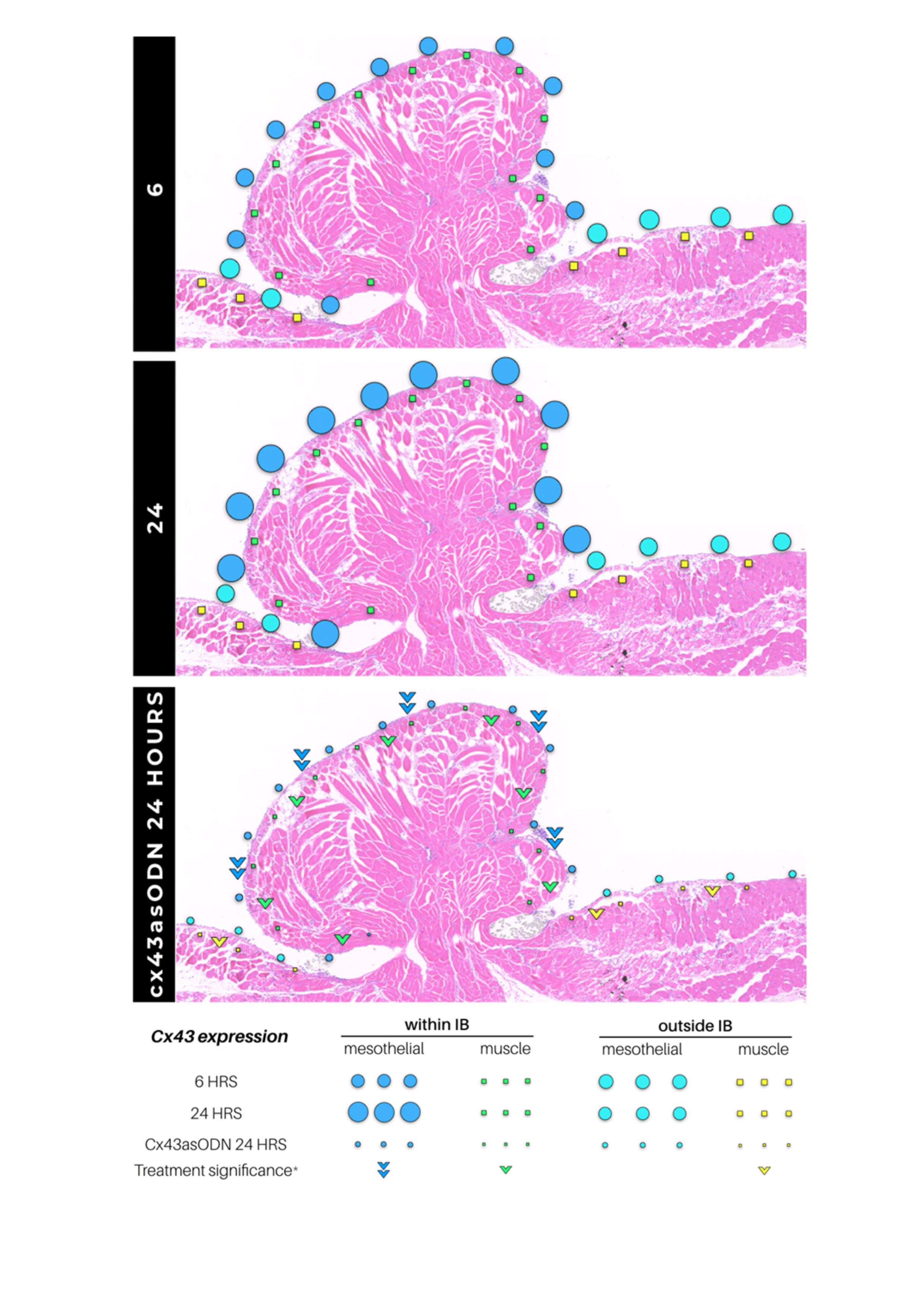

Supplement: Supplementary file 1 [file life-12-01734-s001.zip › Figure S3.tiff]
